# Quality of care in integrated community case management services in Bugoye, Uganda: a retrospective observational study

**DOI:** 10.1186/s12936-018-2241-5

**Published:** 2018-02-27

**Authors:** James S. Miller, Lacey English, Michael Matte, Rapheal Mbusa, Moses Ntaro, Shem Bwambale, Jessica Kenney, Mark J. Siedner, Raquel Reyes, Patrick T. Lee, Edgar Mulogo, Geren S. Stone

**Affiliations:** 1 Massachusetts General Hospital, 55 Fruit St, Boston, MA 02114 USA; 2 University of North Carolina at Chapel Hill School of Medicine, 321 South Columbia St, Chapel Hill, NC 27516 USA; 3 Global Health Collaborative, Mbarara, Uganda; 4 Mbarara University of Science and Technology, Mbarara, Uganda; 5 Bugoye Health Centre, Bugoye Trading Centre, Kasese, Uganda; 6 Lynn Community Health Centre, 269 Union St, Lynn, MA 01901 USA

**Keywords:** Village health workers, Community health workers, Integrated community case management, Quality of care

## Abstract

**Background:**

Village health workers (VHWs) in five villages in Bugoye subcounty (Kasese District, Uganda) provide integrated community case management (iCCM) services, in which VHWs evaluate and treat malaria, pneumonia, and diarrhoea in children under 5 years of age. VHWs use a “Sick Child Job Aid” that guides them through the evaluation and treatment of these illnesses. A retrospective observational study was conducted to measure the quality of iCCM care provided by 23 VHWs in 5 villages in Bugoye subcounty over a 2-year period.

**Methods:**

Patient characteristics and clinical services were summarized using existing aggregate programme data. Lot quality assurance sampling of individual patient records was used to estimate adherence to the iCCM algorithm, VHW-level quality (based on adherence to the iCCM protocol), and change over time in quality of care (using generalized estimating equations regression modelling).

**Results:**

For each of 23 VHWs, 25 patient visits were randomly selected from a 2-year period after iCCM care initiation. In these visits, 97% (150) of patients with diarrhoea were treated with oral rehydration and zinc, 95% (216) of patients with pneumonia were treated with amoxicillin, and 94% (240) of patients with malaria were treated with artemisinin-based combination therapy or rectal artesunate. However, only 44% (44) of patients with a negative rapid test for malaria were appropriately referred to a health facility. Overall, 75% (434) of patients received all the correct evaluation and management steps. Only 9 (39%) of the 23 VHWs met the pre-determined LQAS threshold for high-quality care over the 2-year observation period. Quality of care increased significantly in the first 6 months after initiation of iCCM services (p = 0.003), and then plateaued during months 7–24.

**Conclusions:**

Quality of care was high for uncomplicated malaria, pneumonia and diarrhoea. Overall quality of care was lower, in part because VHWs often did not follow the guidelines to refer patients with fever who tested negative for malaria. Quality of care appears to improve in the initial months after iCCM implementation, as VHWs gain initial experience in iCCM care.

**Electronic supplementary material:**

The online version of this article (10.1186/s12936-018-2241-5) contains supplementary material, which is available to authorized users.

## Background

Village health worker (VHW) programmes undertake a range of different activities in different settings, with varying levels of focus on health promotion, case finding, adherence support, clinical care, and other goals. In many programmes, VHWs provide health education as well as initial assessment and referral to a health facility, but are not trained to administer medical treatment. More recently, interest has increased in equipping VHWs to provide assessment and curative treatment for young children, using variants of integrated community case management (iCCM). In iCCM programmes, VHWs provide diagnosis and treatment of malaria, pneumonia, and sometimes diarrhoea in children under 5 years of age using a standardized assessment and treatment algorithm.

iCCM has the potential to increase access to prompt care, thereby decreasing morbidity and mortality. In addition to its potential benefits, iCCM also carries important risks of harm. Empowering VHWs to provide anti-malarials and antibiotics could result in overtreatment and undertreatment, both of which pose substantial risk. A retrospective observational study was conducted to assess the quality of iCCM care provided by 23 VHWs in five villages over a 2-year period from March 2013–February 2015 in a pilot iCCM programme in Bugoye subcounty, a rural part of Kasese district in Western Uganda.

There are several reasons for further evaluation of iCCM quality of care. First, apart from one study that examined only competence in performing rapid diagnostic tests (RDTs) for malaria [1], prior studies have not assessed the change over time in VHWs’ performance, as they gain greater experience but also become further removed from their initial iCCM training. Second, the decision between iCCM incorporating management of three diseases compared with programmes that focus on a single disease remains controversial given concerns about reduced quality of care [2, 3]. Third, bridging the gap between research-focused programmes and widespread implementation will require approaches to measuring quality of care that require limited time input by staff, particularly clinical staff. The approach demonstrated here, using limited sampling of individual records (via a lot quality assurance sampling approach), helps minimize the cost and staff time needed.

## Methods

### Programme background

The programme described here relies on a wholly volunteer VHW workforce, most of whom have limited formal education, though all are literate in the local language of Lukonjo. VHWs received 3 days of general training and 5 days of iCCM training (in accordance with Ugandan Ministry of Health guidelines), as well as half-day refresher trainings on a quarterly basis. VHWs use the iCCM protocol, called the “Sick Child Job Aid”, to determine the proper care for each patient (Additional file 1). They are equipped with rapid diagnostic tests (RDT) for malaria diagnosis in patients presenting with subjective fever; pneumonia is diagnosed based on age-based respiratory rate cut-offs, and diarrhoea is diagnosed by clinical history. For patients with “danger signs” (evidence of severe illness), VHWs provide initial assessment and referral or accompaniment to a health facility, as well as pre-referral treatment for some conditions. At the discretion of the VHW, caregivers are either given a referral form and instructions to present to the health centre, or are accompanied by the VHW to the health centre in more urgent situations. In this pilot programme, medications for iCCM care were obtained with programme funding and via a separate supply chain, with no reported stock-outs.

VHWs use a “Sick Patient Register” to record each patient they assess. These Sick Patient Registers are submitted monthly and tallied to create a “Monthly Report” for the overall programme. The filed registers and monthly reports provided the data sources for this research.

### Aggregate iCCM service delivery data

Existing aggregate monthly reports (summarizing all patient encounters for VHWs over 2 years) were collected in Microsoft Excel format and combined into a single database. Missing values and outliers were compared with the original paper records, with correction of the database as appropriate. The combined database was summarized using Stata Version 12 (StataCorp, College Station, TX). Descriptive results are reported here to contextualize the quality of care data by describing patient volume.

### Sampling of individual patient visits

A stratified, unweighted sampling design was used to measure quality at the level of individual VHWs, and to provide optimal data for the primary analytic outcome (change over time in quality of care). To ensure a sufficient sample size for this approach, lot quality assurance sampling (LQAS) methods were used to establish the requisite stratum sample size (i.e., number of sampled records per VHW). While originally intended as a rapid and cost-effective quality assurance method for industrial production [4], LQAS has more recently been applied as a monitoring and evaluation tool for a range of health programmes [5], and specifically for data quality in VHW programmes [6–8]. Implementation of lot quality assurance sampling requires specification of upper and lower thresholds for quality, as well as alpha and beta error bounds. Upper and lower thresholds of 0.9 and 0.7, and alpha and beta error bounds of 0.1, were chosen for this study. Using the “LQAS Sampling Plan Calculator” [9], the lot size calculated is 25, with a decision rule of 20, meaning that VHWs with correct patient management of 21/25 (or better) are deemed as providing high-quality care, while those with 20/25 or fewer correct are deemed as not yet providing high-quality care.

VHWs’ Sick Patient Registers were used to create a full sampling frame for each VHW, with 25 patient encounters per VHW randomly sampled. A customized research electronic data capture (REDCap) database was used for data entry, hosted by Partners Healthcare [10]. This database included multiple data validation steps in the user interface to maximize accurate data entry. After data entry, multiple data cleaning steps were performed, including comparison of related variables and examination of outlier values, with comparison to the original paper records.

Logic statements in Stata were used to determine whether each patient received appropriate care according to iCCM protocols. To verify the decision rules and logic statements used for automated determination of iCCM protocol compliance, 125 patient encounters were reviewed and hand-graded using the iCCM protocol, with 100% agreement with the automated determinations.

Disease-specific quality metrics were calculated for the overall programme. Quality metrics included: (1) adherence to correct diagnostic protocol (performing an RDT for malaria for all patients presenting with subjective fever, and measuring a respiratory rate for all patients presenting with cough/fast breathing); (2) appropriate prescribing practices (correctly treating all patients diagnosed with malaria, pneumonia, or diarrhoea; avoiding inappropriate prescriptions for patients not meeting diagnostic criteria); (3) appropriate referral practices in keeping with iCCM guidelines; (4) the proportion of VHWs currently providing high-quality care using the LQAS decision rule cut-off.

Change over time in the quality of iCCM care provided by VHWs was then assessed, to measure how VHWs’ level of experience impacted quality of care. Two competing trends may affect this relationship: VHWs may gain confidence and competence over time in using the iCCM algorithm; alternatively, adherence to the algorithm may decline as VHWs become further removed from their initial iCCM training. Logistic regression models, with a generalized estimating equations (GEE) and robust standard errors to account for correlation by VHW [11, 12], were used to measure the trend in quality of care over time. Each randomly selected patient visit served as a unit of observation. The primary outcome of interest was a dichotomous measure of correct management for each individual patient. The exposure of interest was time since initiation of iCCM care in this programme (defined as date of patient visit minus date of iCCM initiation, March 1, 2013). Initially, the data were graphically depicted to understand the relationship between VHW experience level and quality of care, demonstrating an approximately linear relationship with an inflection point (spline knot) at approximately 6 months after iCCM initiation.

Three main regression models were used to test this hypothesis and optimally explain the relationship of interest: (1) categorizing programme duration into four, 6-month intervals; (2) modelling time as a continuous variable; and (3) modelling time as a continuous variable with a spline knot at 6 months. Quasi-likelihood under the independence model criterion (QIC)—a modification of the Akaike information criterion (AIC) so that it can be applied to GEE models—was used to assess goodness of fit of the two continuous models [13]. Finally, post-estimation margins were used to estimate and compare the proportion of visits with correct management predicted by these models at different time points.

## Results

### Aggregate iCCM service delivery data

#### Patient population and summary of care provided

In the overall iCCM pilot programme, 23 VHWs in 5 villages completed 5462 patient visits over 2 years; 2102 (38%) patients received evaluation/treatment within 24 h (Table 1). There were 2887 (53%) patients with fever, 2276 (42%) with subjective cough/fast breathing, and 1460 (27%) with diarrhoea (percentages add to more than 100% since some patients presented with more than one complaint). There were 2431 malaria diagnoses by RDT over the 2-year period (thus, 45% of all patients seen were diagnosed with malaria), with 1023 negative RDTs (thus, 70% of RDTs performed were positive). There were 2380 (44%) patients treated with oral artemisinin combination therapy (ACT), and 36 (0.7%) treated with rectal artesunate. The aggregate data do not distinguish between patients presenting with cough and patients with confirmed elevated respiratory rate; thus, it was not possible to determine the number of patients diagnosed with presumed pneumonia. However, 2370 (43%) patients received amoxicillin. For diarrhoea treatment, 1555 (28%) patients were treated with ORS, and 1554 (28%) treated with zinc. There were 560 (10%) patients referred to a health centre, 0 medication reactions reported, and 2 (0.04%) deaths reported (one case was found in the LQAS sample in which a medication reaction occurred, so the reporting of 0 medication reactions in all patient visits must be due to an error in data aggregation.).

**Table 1:** Summary of patient demographics and care provided for all iCCM patients (over 2 years)

Characteristic	Overall n (%)	Per VHW mean (range)
Total patient visits	5462 (100%)	237 (105–441)
Presenting complaints^a^		
Fever	2887 (53%)	126 (43–316)
Cough/fast breathing	2276 (42%)	99 (40–221)
Diarrhoea	1460 (27%)	63 (18–139)
Patients with danger signs^b^	90 (2%)	3.9 (0–16)
Patients receiving RDT for malaria	3454 (63%)	150 (56–343)
Positive RDTs	2431 (70%)	106 (19–265)
Negative RDTs	1023 (30%)	44 (5–102)
Patients receiving oral ACT	2380 (44%)	103 (17–255)
Patients receiving rectal artesunate	36 (0.7%)	1.6 (0–10)
Patients receiving oral amoxicillin	2370 (43%)	103 (41–244)
Patients receiving ORS	1555 (28%)	68 (23–138)
Patients receiving zinc	1554 (28%)	68 (22–140)
Patients treated with 24 h of illness onset	2102 (38%)	91 (0–319)
Patients referred to health centre	560 (10%)	24 (1–80)
Medication reactions^c^	0 (0%)	0 (n/a)
Deaths	2 (0.04%)	0.09 (0–1)

^a^Percentages add to > 100%, as some patients presented with multiple complaints

^b^95 danger signs patients were identified in the sick patient registers, indicating that five patients were missed in the aggregate data

^c^Given that one case was found in the LQAS sample in which a medication reaction occurred, the reporting of zero medication reactions in all patient visits must be incorrect

Each VHW saw an average of 237 patients (range 105–441)—an average of 126 presenting with fever (range 43–316), 99 with cough/fast breathing (range 40–221), and 63 with diarrhoea (range 18–139); see Table 1. Each VHW performed an average of 106 positive RDTs (range 19–265) and 44 negative RDTs (range 5–102). On average, each VHW prescribed ACT 103 times (range 17–255), amoxicillin 103 times (range 41–244), oral rehydration solution (ORS) 68 times (range 23–138), and zinc 68 times (range 22–140).

### Sampling of individual patient visits

#### Patient population and summary of care provided

Of the 575 patient visits sampled, 52% (286) of patients were female and 48% (265) were male, with a mean age of 31 months (range 2 months–5 years); see Table 2. Due to a lack of standardization in reporting, VHWs recorded patients’ ages using inconsistent units (weeks, months, or years). For the purposes of analysis, all reported ages were converted to months, which will tend to underestimate actual ages. Of note, there were 66 (11%) patients in this sample who were 5 years old at the time of the visit, whereas the iCCM algorithm is intended only for children under 5 years of age. This may result from ambiguity in the “Sick Child Job Aid”, which lists age ranges that appear inclusive of 5 year olds (i.e., “children 1–5 years”). Given this ambiguity, we did not treat this as an error by VHWs.

**Table 2:** Patient demographics and presenting conditions for LQAS sample (overall n = 575)

Characteristic	N (%) or mean (range)
Female	286 (52%)
Age	31 months (2 months–5 years)
Village	
Bugoye	125 (22%)
Ihani	112 (19%)
Kanyaminigo	125 (22%)
Kikokera	88 (15%)
Muramba I	125 (22%)
Presenting complaints^a^	
Fever	293 (51%)
Cough/fast breathing	237 (41%)
Diarrhoea	155 (27%)
Other	66 (11%)
Patients with danger signs	11 (2%)
Outcomes	
Patients referred to health centre	66 (11%)
Medication reactions	1 (0.2%)
Deaths	0 (0%)

^a^Percentages add to > 100%, as some patients presented with multiple complaints

In regard to presenting complaints and outcomes, 293 (51%) patients presented with fever, 237 (41%) with cough/fast breathing, and 155 (27%) with diarrhoea (see Table 2; percentages add to more than 100% as some patients presented with multiple complaints). Only 11 (2%) patients were recorded as presenting with “danger signs.” There were 66 (11%) patients referred to a health facility. Only 1 (0.2%) medication reaction was recorded. There were no recorded deaths among the 575 sampled patient visits.

#### Quality of care—descriptive results

##### Fever/malaria care

Of the 293 patients presenting with fever, 283 (97%) correctly received an RDT for malaria. Additionally, there were 73 patients who did not have fever recorded as a presenting complaint, but who nonetheless received an RDT. Of 255 patients with a positive RDT for malaria, 240 (94%) received correct management (Table 3; this includes patients for whom fever was not recorded as a presenting complaint, but who nonetheless received an RDT and were found to have malaria). There were 101 patients with a negative RDT, of whom only 44 (44%) received correct management (referral, with concomitant pre-referral treatment if dangers signs were present); 56 patients with a negative RDT were not referred to a health centre (of whom three also incorrectly received oral ACT), and one patient with a negative RDT and danger signs did not receive appropriate pre-referral treatment. Overall, 11 patients who did not have a positive RDT received ACT, out of 250 ACT prescriptions; thus, 4% of ACT prescriptions were not indicated.

**Table 3:** Quality of care in LQAS sample

Measure	N (%)
RDT performed for patient presenting with fever (n = 293)	283 (97%)
Malaria patients receiving correct management (n = 255)	240 (94%)
Patients with negative RDT receiving correct management (n = 101)	44 (44%)
Respiratory rate recorded for patient presenting with cough/fast breathing (n = 237)	223 (94%)
Patients with elevated respiratory rate receiving correct treatment (n = 228)	216 (95%)
Patients with diarrhoea receiving ORS and zinc (n = 155)	150 (97%)
Patients inappropriately treated with ACT (n = 575)	11 (2%)
Patients inappropriately treated with amoxicillin (n = 575)	23 (4%)
Patients inappropriately treated with ORS, zinc, or both (n = 575)	18 (3%)
Patients with danger signs appropriately referred to health centre (n = 11)	11 (100%)
Patients with danger signs receiving appropriate pre-referral treatment (n = 11)	4 (36%)
Patients receiving overall correct management (n = 575)	434 (75%)
Patients receiving overall correct management in months 1–6 of iCCM implementation (n = 152)	96 (63%)
Patients receiving overall correct management in months 7–12 of iCCM implementation (n = 146)	116 (79%)
Patients receiving overall correct management in months 13–18 of iCCM implementation (n = 154)	117 (76%)
Patients receiving overall correct management in months 19–24 of iCCM implementation (n = 123)	105 (85%)
VHWs providing high-quality care over 2 years, according to LQAS decision rules (n = 23)	9 (39%)

##### Cough/fast breathing/pneumonia care

Of 237 patients who presented with cough/fast breathing, 223 (94%) correctly had their respiratory rate recorded. Of the 228 patient visits in which an elevated respiratory rate was documented, 216 (95%) were treated appropriately with amoxicillin (Table 3). Additionally, 23 patients for whom a respiratory rate was not recorded, or who did not meet criteria for elevated respiratory rate, were treated with amoxicillin (out of 239 amoxicillin prescriptions); thus, 10% of amoxicillin prescriptions were not indicated.

##### Diarrhoea care

Of 155 patient visits with diarrhoea as a recorded complaint, 150 (97%) were managed appropriately with ORS and zinc (Table 3). Additionally, 18 patients for whom diarrhoea was not recorded as a presenting complaint were nonetheless treated with ORS or zinc; thus, 12% of prescriptions were not indicated.

##### Care for patients with “danger signs”

Of 11 patient visits with “danger signs” recorded, all 11 were documented as having been appropriately referred to a health centre, but only four had appropriate pre-referral treatment documented (Table 3). However, the lack of full clinical documentation in the current record system and small number of patients limit our analysis.

##### Overall quality of care

After combining all the decision rules for correct management, over the 2-year period studied 75% (434) of patient visits received correct evaluation and treatment. The proportion of patient visits with correct management was lowest in months 1–6 of the iCCM pilot, with 63% (96) of patients managed correctly, compared with 79% (116) in months 7–12, 76% (117) in months 13–18, and 85% (105) in months 19–24. This trend was driven substantially by improved performance in the treatment of malaria and pneumonia. In months 1–6, VHWs followed appropriate malaria treatment protocols in 77% (51) of relevant visits, compared with 93% (74) in months 7–12, 97% (66) in months 13–18, and 94% (50) in months 19–24. Similarly, in months 1–6 VHWs followed appropriate pneumonia treatment protocols in 73% (52) of visits, compared with 91% (48) in months 7–12, 91% (62) in months 13–18, and 92% (54) in months 19–24.

At the individual VHW level, according to the LQAS decision rule cut-off of 20 patients (out of 25), 9 VHWs are classified as providing high-quality care over the 2-year period, while 14 are classified as not yet providing high-quality care. The median proportion of patient visits managed correctly by each VHW was 80%, with a range of 32–92%.

#### Quality of care over time—logistic regression models

**Fig. 1:**
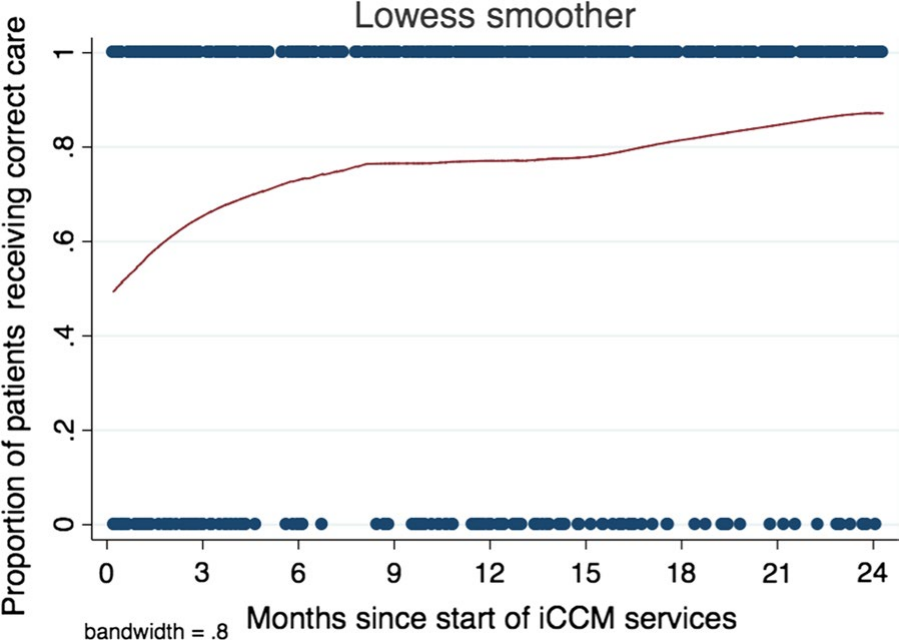
Correct management over time—Lowess plot

**Fig. 2:**
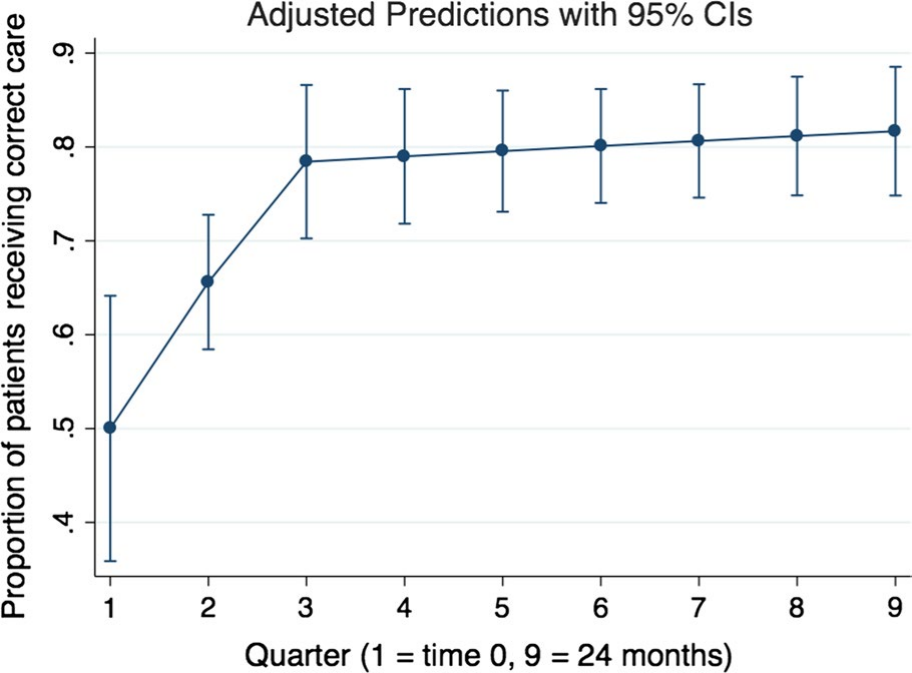
Post-estimation margins plot of regression model with spline knot at 6 months

Graphical depiction of the data identified a trend toward improved quality of care during the first 6 months after initiation of iCCM care, and a levelling off of the relationship thereafter (Fig. 1). The first model, categorizing time into four 6-month periods, revealed significantly higher odds of correct patient management in months 7–12, 13–18, and 19–24, compared with months 1–6 (p = 0.003, p = 0.032, and p = 0.001, respectively; Table 4). Modelling time as a continuous variable estimated a 6% increased odds of correct management for each additional month since iCCM initiation (OR = 1.06, 95% CI 1.02–1.09, p < 0.001). However, the addition of a spline knot at 6 months demonstrated a significant increase in the proportion of appropriate management per month in the first 6 months (OR = 1.24, 95% CI 1.08–1.43, p = 0.003), but not for months 7–24 (OR = 1.01, 95% CI 0.98–1.04, p = 0.47; Table 4 and Fig. 2). The QIC estimate comparing the continuous and spline models demonstrated an improved model fit after the addition of the spline term (QIC decreased from 614.6 to 612.2). Using post-estimation margins, the model with a spline term predicts an initial proportion of patients managed correctly of 50% (95% CI 36–64%), increasing to 78% (95% CI 70–87%) at 6 months, 80% (95% CI 73–86%) at 12 months, 81% (95% CI 75–87%) at 18 months, and 82% (95% CI 75–89%) at 24 months (see Fig. 2). The strength of association was similar with and without the inclusion of a quasi-outlier (one VHW with substantially lower performance than all others).

**Table 4:** GEE logistic regression models for overall quality of care over time

Variable	OR	95% CI	p value	Model QIC^a^
Model 1: biannual groupings, with months 1–6 as reference group				n/a
Months 7–12	2.33	(1.33, 4.11)	p = 0.003	
Months 13–18	1.84	(1.05, 3.20)	p = 0.032	
Months 19–24	3.05	(1.76, 5.29)	p < 0.001	
Model 2: time as a continuous variable				614.62
Months since iCCM services initiation	1.06	(1.02, 1.09)	p < 0.001	
Model 3: time as a continuous variable, with a spline knot at month 6				612.16
Months since iCCM services initiation—months 1–6	1.24	(1.08, 1.43)	p = 0.003	
Months since iCCM services initiation—months 7–24	1.01	(0.98, 1.04)	p = 0.47	

^a^Quasi-likelihood under the independence model criterion (QIC). This is a modification of the Akaike information criterion (AIC) so that it can be applied to GEE regression models to assess goodness of fit of different models. A lower QIC term reflects a better-fitting regression model. It is not applicable when using factor variables, so it is not calculated for Model 1, which uses a categorical time variable

## Discussion

In this study, quality of care was high for the core steps involved in managing uncomplicated malaria, pneumonia, and diarrhoea. These results generally accord with prior research. A recent systematic review of malaria care documented very high quality of care by VHWs, with 95–100% correct interpretation of RDTs, and appropriate treatment of patients with positive RDTs in over 90% of cases [14]. Quality of care for pneumonia has been lower in prior studies, due to difficulty in measuring respiratory rate and in following the iCCM algorithm for pneumonia, though some programmes have demonstrated high-quality care. A systematic review of 9 programmes in which VHWs managed both malaria and pneumonia described a median of 75.8% of patients diagnosed with pneumonia who received appropriate antibiotic treatment [3]. By comparison, the higher quality for pneumonia care reported in this study should be interpreted with caution, as these methods do not assess VHWs’ ability to measure respiratory rate appropriately. Prior evidence on quality of care for diarrhoea is more limited, but shows relatively high quality, with a recent study from Uganda documenting 88.6% of patients receiving correct management [15], while another study in Ethiopia study reported 79% correct management [16], and two studies in Malawi showed 90% [17] and 69% [18] correct management.

This study shows much lower adherence to referral guidelines for patients with fever who had negative RDTs; indeed, this was the single most common error made by VHWs, comprising nearly 40% of the patient visits with incorrect management. Similarly, prior studies have shown low adherence to referral guidelines, with only 18.2–47.1% of patients receiving appropriate referral [14]. This error might result from a misunderstanding of the iCCM protocol, a belief that these families are unlikely to visit a health centre if referred, a reluctance to refer patients who are relatively likely to have a self-limiting viral infection, or some other reason. Further elucidation of VHW perspectives is needed to understand the reasons for this error. Of note, in some versions of the iCCM algorithm used by other programmes, patients presenting with fever who have a negative RDT (and no danger signs or fast breathing) receive symptomatic management and follow-up in the community rather than referral to a health centre, with high rates of spontaneous improvement, suggesting that failure to refer these patients may be a relatively low-consequence error [19].

Inappropriate use of anti-malarials and antibiotics (i.e., use when not indicated by the iCCM protocol) was relatively low in this study, with 4% inappropriate use of ACT and 10% inappropriate use of amoxicillin. These rates are slightly higher than in a recent study in Ethiopia [16], and broadly comparable to a multi-site trial in Ghana, Burkina Faso, and Uganda, which found higher levels of overuse in Ghana and Burkina Faso, but very low levels of overuse in Uganda [20]. As a point of comparison, clinical staff at health centres in Uganda appear to have far higher rates of inappropriate antibiotic use than the VHWs in Bugoye or the other iCCM programmes discussed above [21].

The LQAS results demonstrate substantial variation in quality among VHWs. As a monitoring tool, this may offer a useful means of identifying VHWs who would benefit from additional training and mentorship. While the final LQAS result, in which only 9 of 23 VHWs are classified as providing high-quality care, may seem disconcerting, the analytic results of this study document a significant trend toward improvement in quality in the initial months of iCCM implementation. Due to the sample size, the LQAS assessment could not be repeated in a rigorous way using only months 7–24 to compare these results. Similarly, the LQAS assessment could not be repeated without the inclusion of RDT-negative patients who were not referred.

The analytic results of this study demonstrate an encouraging improvement over time in VHWs’ competence during the initial 6-month period after iCCM implementation, increasing from approximately 50–80% correct management rates, after which point the proportion of patients managed appropriately seemed to plateau.

## Limitations

This study has a number of limitations, in part resulting from our use of routine clinical records as the data source. First, and perhaps most importantly, record review assesses quality of documented care, which may differ from the quality of actual care provided. Additionally, only limited validation of VHWs’ documentation has occurred in this programme. The methods used here cannot assess whether VHWs performed RDTs correctly or measured respiratory rate accurately, so in that respect these results may overestimate quality of care. Conversely, in a programme in which some VHWs have limited literacy skills, some patients may have received correct care but incorrect documentation. For instance, patients who received ORS or zinc but did not have diarrhoea listed as a presenting complaint are considered to have received incorrect management, even though this may have resulted from a record-keeping error. Prior research comparing record review (as employed here) with direct observation of VHWs and re-examination of patients by a trained clinician suggests that the two methods yield broadly similar results, with record review overestimating quality for some metrics [17].

Second, not all relevant dimensions of quality can be assessed with the available data, such as correct dosing of medications or appropriate instructions for caretakers. Likewise, since the specific “danger sign” is not recorded on the clinical form used, it is impossible to characterize fully the management of this subset of patients, who likely face the highest risk of morbidity and mortality. The available data also do not provide any information on completion of referrals.

Third, for the analytic outcome of change in quality over time, the small number of VHWs in the iCCM pilot programme as well as our data structure prevent further analysis of other factors, such as education level or patient volume. Additionally, there may be important trends in quality of care over time that arise outside the 2-year period observed here.

Finally, due to the lack of a reliable unique identifier, it is possible that two different illness episodes for the same individual are included in the sample of patient visits. However, the impact of this issue is likely small, since the two visits for a given patient are not necessarily correlated with respect to quality of care, and since a GEE model with robust standard errors was used to account for non-independence of the data.

## Conclusions

Despite its limitations, this study yields several significant practical results. First, it provides initial results on the trend in quality of iCCM care over time—a topic highly relevant to the training and supervision needs of VHWs as iCCM programmes expand in Uganda and elsewhere. Further evaluation may clarify the impact of patient volume on this trend, with implications for the size of the catchment area assigned to each VHW. Further research using a larger sample size would also allow for evaluation of VHW-level factors such as level of formal education completed. Second, from a practical standpoint, it demonstrates the feasibility of measuring iCCM quality of care using routine data. LQAS requires additional data entry, but the relatively small sample sizes make it less costly than more exhaustive data entry of all patient encounters. Third, this research demonstrates opportunities for the Ugandan Ministry of Health to improve iCCM protocols and training by identifying common errors. Fourth, on a broader level, it bolsters the evidence for iCCM programmes given the relatively high quality of care for uncomplicated malaria, pneumonia, and diarrhoea, as well as the trend toward improvement over time.

## Additional file

**Additional file 1:** The Republic of Uganda Ministry of Health Sick Child Job Aid (translated in English and Lukonjo for use in Bugoye).

## Abbreviations

ACT: artemisinin-based combination therapy
GEE: generalized estimating equations
iCCM: integrated community case management
LQAS: lot quality assurance sampling
ORS: oral rehydration solution (also called oral rehydration salts)
QIC: quasi-likelihood under the independence model criterion
REDCap: research electronic data capture
RDT: rapid diagnostic test (for malaria)
UNICEF: United Nations Children’s Fund
VHW: village health worker
WHO: World Health Organization
