# Supplementary material for: Quality of care in integrated community case management services in Bugoye, Uganda: a retrospective observational study
Source: Malar J. 2018 Feb 27;17:99. doi: 10.1186/s12936-018-2241-5 (PMC6389111; doi:10.1186/s12936-018-2241-5)
Supplement: Supplementary file 1 — Additional file 1: The Republic of Uganda Ministry of Health Sick Child Job Aid (translated in English and Lukonjo for use in Bugoye). [file 12936_2018_2241_MOESM1_ESM.pdf]

1. ASK THE CAREGIVER / BULHAYA OYULYALETHA OMWANA

Name of Child / Erina ly'omwana ____________________

How old is the child? Omwana awithe emyaka mingahi?

Age / Buthuku bungahi ☐

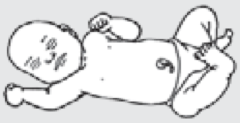

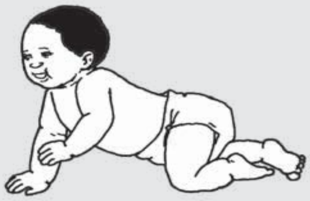

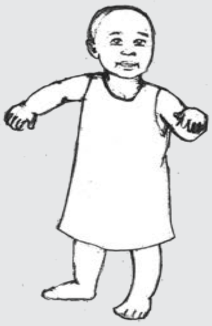

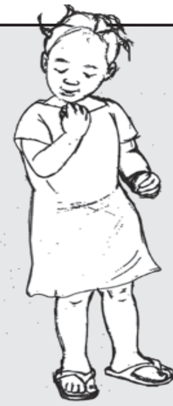

☐ **0-7 days/ ebiro** ☐ **2-11 months/ emighesera** ☐ **1-2 years/ emyaka** ☐ **3-5 years/ emyaka**

2. ASK CHILD'S PROBLEMS / BULHAYA EBITSIBU EBYOMWANA AWITHE

Does the child have cough, diarrhoea or fever?
Omwana anemukoholha, erihurungana, kwitse erihurunganako mubiri?

If yes, for how long? / Amabugha athi eee……….. Amaghunza buthukuchi?

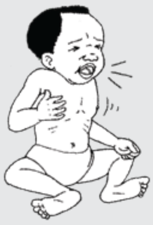

☐ **Cough / Erikoholha**
Number Of Days / Omughanzo w'ebiiro ☐

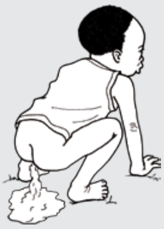

☐ **Diarrhoea / Erihurungana**
Number Of Days / Omughanzo w'ebiiro ☐

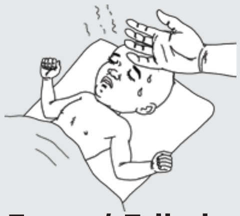

☐ **Fever / Erihuhana komubiri**
Number Of Days / Omughanzo w'ebiiro ☐

If **Fever** present do a RDT.

Omwana amabya inianemuhuhana komubiri iwapima omutsutsa omwa kabatsi kano [RDT].

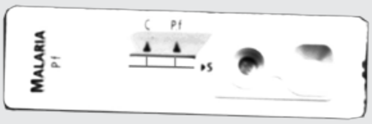

Positive ☐ +

Negative ☐ -

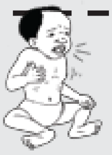

If **Cough** is present, check for **Fast Breathing**.
Omwana amabya inia ni'mukoholha, iwalebya nganemuhumulha.

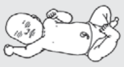

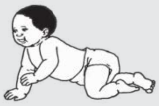

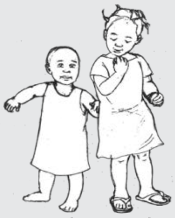

0 - 7 days / ebiro	2-11 months/ emighesera	1-5 years / emyaka
**60 or more** breaths per minute	**50 or more** breaths per minute	**40 or more** breaths per minute
**60 kwitse erilhaba** Emirundi ey'erihumulha omwa dakika	**50 kwitse erilhaba** Emirundi ey'erihumulha omwa dakika	**40 kwitse erilhaba** Emirundi ey'erihumulha omwa dakika
Number Of Breaths / Emirundi eyerihumulha ☐	*Number Of Breaths / Emirundi eyerihumulha* ☐	*Number Of Breaths / Emirundi eyerihumulha* ☐

SICK CHILD JOB AID /
EKYERIGHENDERAKO OKWITHAMBIRA OMWANA OYULHWERE

3. ASK AND LOOK FOR DANGER SIGNS AND REFER / BULHAYA KANDI USAMALIRE OMWANA NGANEKWEBISOSEKA NO EBYERITHOHA NERYO IWAMUTHUMA OKWITHAMBI RIRO

Refer to Step 4a because children with some of these danger signs need pre-referral treatment.

Suba okwidara lyakani (4a) ahabwa eribya omwana awithe othundi othuminyikalho oth'obulhwere othukalhangirawa othutholere obubalya

Any child or newborn with...
Omwana kwitsi olhumekeke eyiwithe bino...

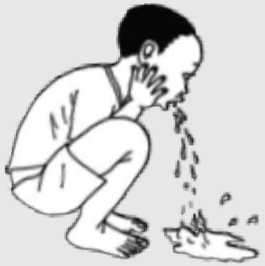

Vomiting ☐
Vomits everything

Erisalha
Erisalha obulikalyo kosi

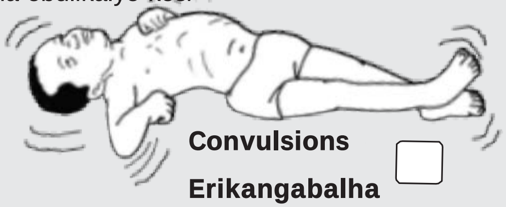

Convulsions ☐

Erikangabalha

Chest in-drawing ☐

Ekikuba ekyo mwana erisuba omwakathi kutsibu akahumulha

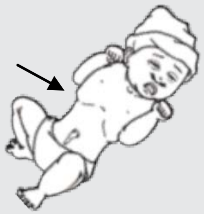

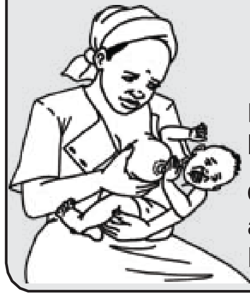

Not able to breastfeed or drink ☐

Omwana abirighana amabere kwitse erinywa

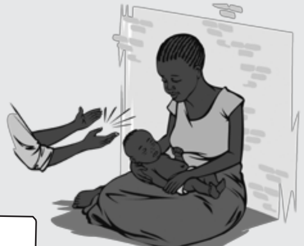

Very sleepy or unconscious/difficult to wake ☐

Omwana akasiba omwathulho kwitse abirithoha

A newborn with...
Olhumekeke nabino...

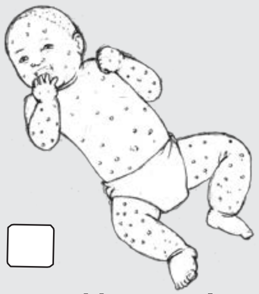

☐

Many skin pustules

Omubiri owo lhumeke owali kwesvonvanva

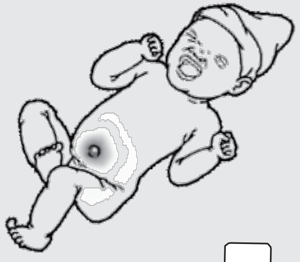

☐

Infected umbilical cord

Engundi yomwana eyikalhwa mwamahira

A child with...
Omwana nabino ...

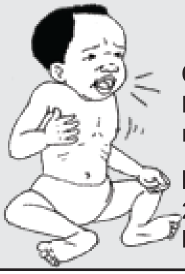

Cough ☐
For 21 days or more

Erikoholha
21 [Ebiro] kwitse erilhaba

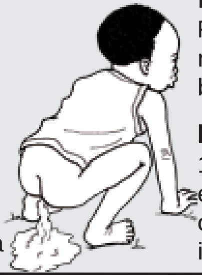

Diarrhoea ☐
For 14 days or more, or with blood

Erihurungana
14 [Ebiro] kwitse erilhaba kandi omwabubi immune omusasi

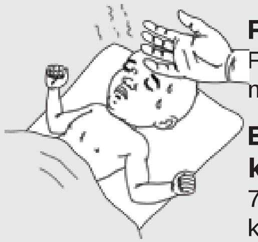

Fever ☐
For 7 days or more

Erihuhana komubiri
7 [Ebiro] kwitse erilhaba

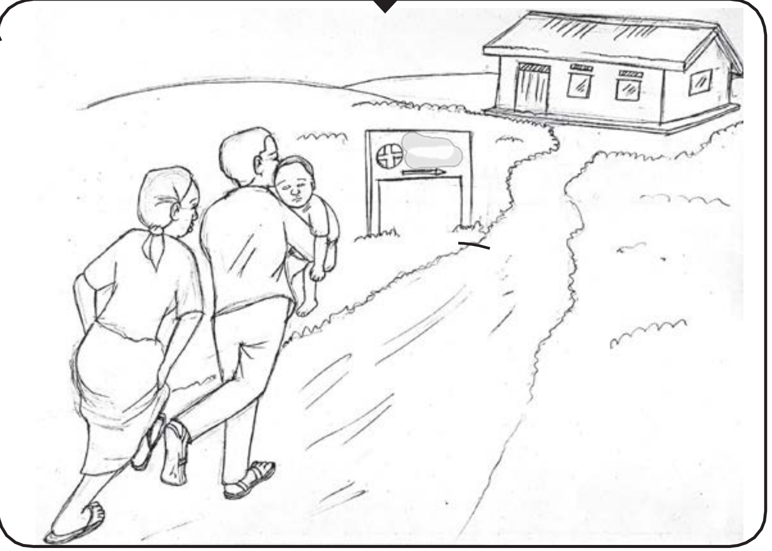

Go immediately to the nearest Health Centre.

Iwaghenda lhuba okwitha mbiriro eririhakuhi naghu.

4a. PRE-REFERRAL TREATMENT / OBUBALYA OBOWANGAHA OMWANA OYULHOHIRE ATHALYAGHENDA OKWITHA MBIRIRO

If the child has any of the danger signs below, give the following treatment:
Omwana amabya n'obuminyikalho obukalhangirawa ob'obulhere obunene buno, muhe omubatsi ono

Chest In-drawing or Fast Breathing
With a Danger Sign
Ekikuba erisuba omwakathi kutsibu akahumulha kwitse erihumulha lhubalhuba
Awithe akaminyikalho akalhangirawa ak'obulhwere obunene

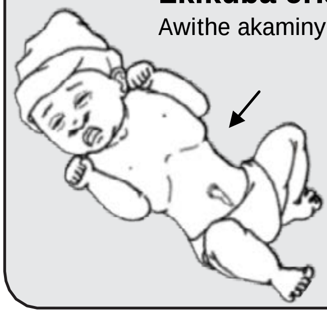

☐ Give first dose of Amoxycilin before you refer.
Iwahaebinini ebya Amoxycilin wuthe wathuma omwana okwithambiriro.

2 - 11 months / emighesera: 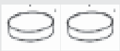 2 tabs from RED PACK
Ebinini bibiri erilhwa omwa ka bokisi ako musasi

1 - 5 years / emyaka: 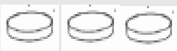 3 tabs from GREEN PACK
Ebinini bisathu erilhwa omwakabokisi ake kithi kibisi

Diarrhoea
For 14 days or more, or with blood in the stool
Erihurungana
14 [Ebiro] kwitse erilhaba kandi omwabubi immune omusasi

☐ Begin giving the child ORS before you refer. Advise caregiver to continue giving ORS to the child on the way to the health centre.

Iwaha omwana yeritere kandi iwabwira omuthwaniri erighenda embere inianimuhere rya omwana yeritere omwanzira

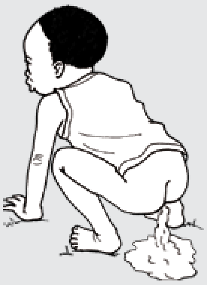

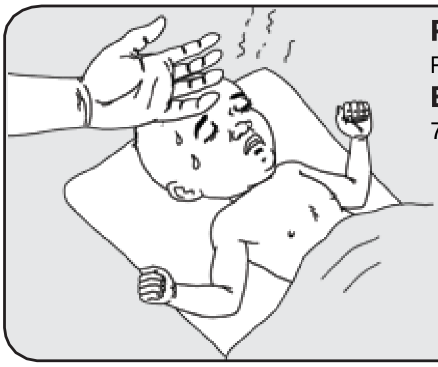

Fever
For 7 days or more
Erihuhana komubiri
7 [Ebiro] kwitse erilhaba

☐ Give first dose oral Anti-Malarial ACT.
Iwahererya omubatsi owo mutsutsa.

4 months - 2 years/
4 emighesera – 2 emyaka: 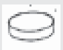 1 tab from YELLOW PACK
Ekinini kighuma kisa erilhwa omwa kabokisi ake kisande

3-5 years/emyaka: 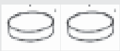 2 tabs from BLUE PACK
Ebinini bibiri erilhwa omwa kabokisi ake bururu

Fever and a General Danger Sign

Erihuhana komubiri haima nekisosekano ekyerithoha

☐ Give Rectal Artesunate.
Iwahererya omubatsi owo mutsutsa erilhabya welhuhende

4 - 11 months / emighesera: 1 cap / Akanini kaghuma

1 - 3 years / emyaka: 2 caps / Ebinini bibiri

4-5 years / emyaka: 4 caps / Ebinini bini

☐ Help caregiver give dose.
Wathikaya omuthwaniri erihererya omubatsi.

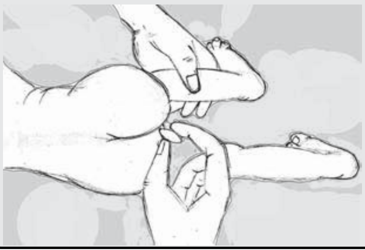

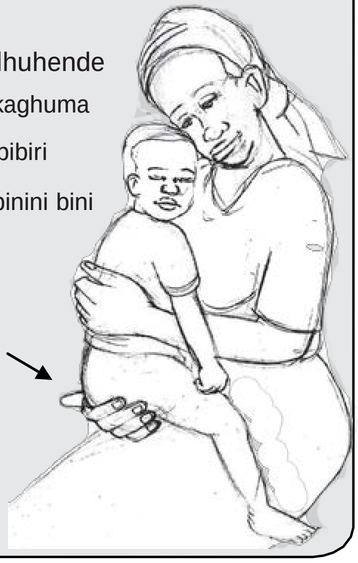

Newborn
With a Danger Sign

Olhumekeke nakaminyikalho akerithoha

☐ Refer
Iwamuthuma okwithambiriro

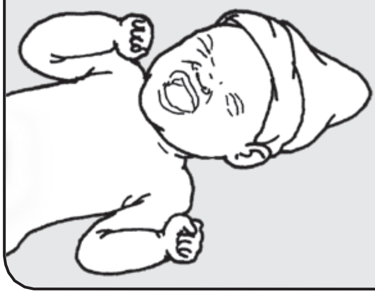

4b. TREAT AND ADVISE / THAMBIRA NERIHABULHA

If the child has any of the symptoms below with NO danger signs, give the following treatment:

Omwana amabya isyawithe othuminyikalho othweriyibughira kandi isyawithe othuminyikalho othukalhangirawa oth'obulhwere obunene, muhe omubatsi ono.

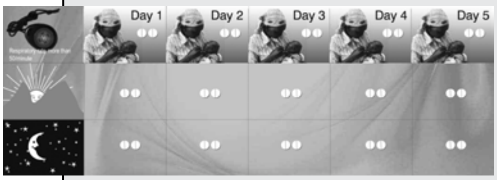

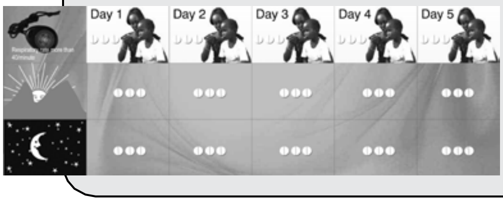

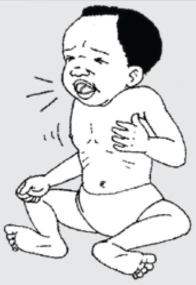

Cough with fast breathing for less than 21 days
Erikoholha erithelya ghunza ebiro abiri nakiyima [21]

- ☐ Give oral antibiotic – Amoxicillin.
  Hereraya ebinini ebyeri mera [Amoxycilin]
- ☐ Advise caregiver to give / Habulha omuthwaniri erihererya bino:

2-11 months / emighesera:		2 tabs from RED PACK<br>Ebinini bibiri erilhwa omwa kabokisi oko musasi
1-5 years / emyaka:		3 tabs from GREEN PACK<br>Ebinini bisathu erilhwa omwa kabokisi ake kithi kibisi

- ☐ Help caregiver give first dose now.

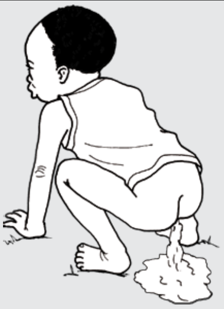

Diarrhoea for less than 14 days, and without blood in stool
Erihurungana ahisi yebiro 14 kandi simuli musasi omobubi

- ☐ **Give ORS.** Help caregiver give child ORS solution in front of you until the child is no longer thirsty.
  Hereraya eritere. Wathikaya omuthwaniri erihererya eritere emberesyaghu aho omwana akendireke ra erikwa enyotha
- ☐ **Give caregiver 2 ORS packets to take home**. Advise caregiver to give as much as child wants, but at least ½ cup ORS solution after each loose stool.
  Hereraya omuthwaniri yobusa ho bubiri obwe eritere. Habulha omuthwaniri erihererya omwana ngoko kayisunga, hachiri embindi eyekikopo obuli hurungana
- ☐ **Give zinc supplement.** Give 1 dose daily for 10 days.
  Hereraya omwana yokabatsi [Zinc] obulikiro habwe ebiro ikumu [10]

2 to 6 months/ emighesera:		½ tab (Total 5 tabs) / Embindi eyekinini [5]
6 months/emighesera to 5 years/emyaka:		1 tab (Total 10 tabs) / Ekinini kighuma [10]

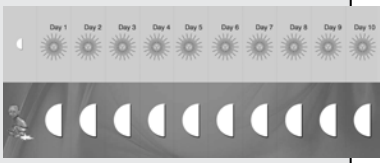

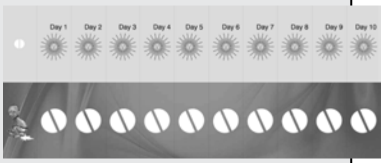

- ☐ Help caregiver give first dose now.
  Wathikaya omuthwaniri erihererya omubatsi

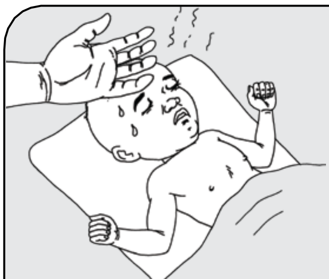

Fever less than 7 days
Erihuhana komubiri Ahasi yebiro musanju [7]

- ☐ Do a RDT, if positive give Oral Anti-Malarial ACT. If negative advise and refer to the Health Center.
  Piima Omutsutsa, amabya inianemo hereraya omubatsi womutsutsa [ACT].
  Amabva athemo, habulha kandi wumuthwale okwithambi riro eririhakuhi nawu.
- ☐ Give 1 dose twice daily for 3 days.
  Hereraya ekinini kighuma kabiri ekiro habwe ebiro bisathu

4 months/emighesera to 3 years/ emyaka:		1 tab from YELLOW PACK<br>Kinini kighuma erilhwa omwa kabokisi ekekisande
3 - 5 years/emyaka:		2 tabs from BLUE PACK<br>Ebinini bibiri erilhwa omwa kabokisi ekeburu

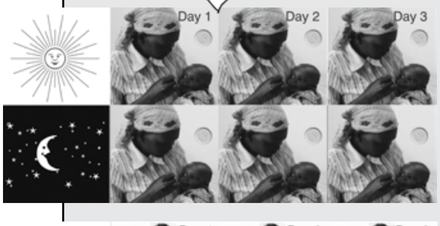

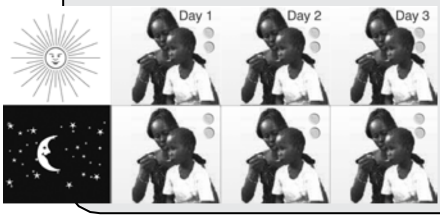

- ☐ Help caregiver give first dose now.
  Wathikaya omuthwaniri erihererya omubatsi
- ☐ Advise caregiver on use of a bed net (LLIN).
  Habulha omuthwaniri okwi kolesya akathimba akemibwa

5. ADVICE FOR ALL CHILDREN TREATED AT HOME / ERIHABULHWA ERYABANA BOOSI ABAKATHAMBIRIRAWA

Give more fluids and continue feeding.
Hereraya ebyerinywa binene kandi nerighenda embere neryongya.

☐ Advise caregiver to give plenty of homemade fluids such as:
Habulha omuthwaniri okwi hererya ebyerinywa ebikakolerawa omwaka ngabino:

- Clean water / Amaghestse awaheniriye
- Soup / Omukubi
- Yoghurt drinks / Ekyerinywa ekikalhwa omwamathe awakwire
- Light porridge / Obusara obwanguhire
- and give ORS / Kandi hereraya eritere

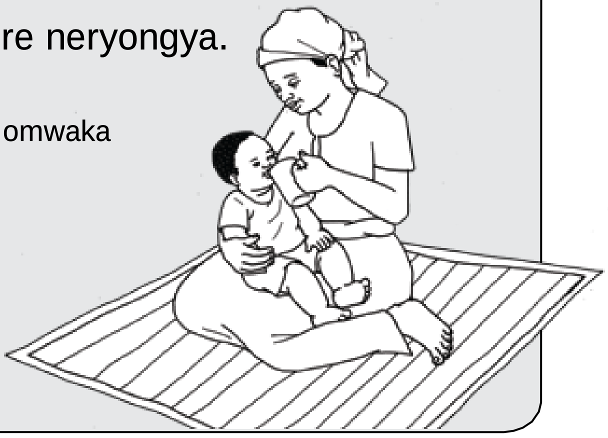

Go to the health facility if...
Ghenda okwithambiriro habwo...

☐ Advise to go to the health facility if the child:
Habulha erighenda okwithambiriro omwana amabya

- Cannot drink or feed / Isyanganywa kwitse eririya
- Has blood in stool / Awithe omusasi omobubi
- Becomes sicker / Amalhwalha kutsibu
- Develops any other danger sign / Amakangania akasosekano akerilhwalha kutsibu
- Has not improved in 2 days / Syathahingisya omobiro bibiri

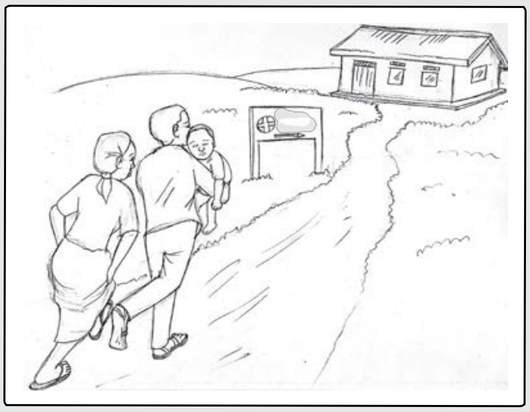

Sleep under a net
Kesaya ahisi esyakathimba akemibwa

☐ Advise caregiver to be sure the child sleeps under a mosquito net.
Habulha omuthwaniri erilhangira indi omwana akakesaya ahisi esya kathimba akemibwa.

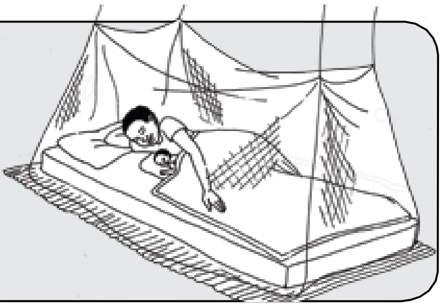

Follow up child in 3 days.
Kwama omwana omo biro bisathu [3]

☐ Decide if you (the VHT member) will go to the child or if the caregiver will bring the child to you.
Wuthwemo iwe ngomuthahwa erighenda eyiri omwana kwitse omuthwaniri erikulethera omwana

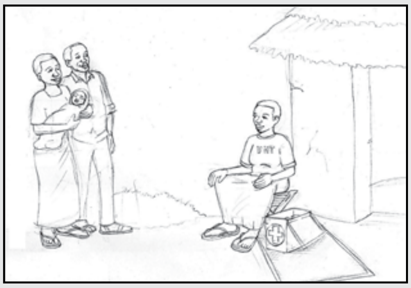

6. ROUTINE CARE FOR THE NEWBORN / OBUWATHIKYA OBWA BULIKIRO OBYO LHUMEKEKE

Keeping the baby warm.
Eribika omwana eribya inianahuhire

Advise the caregiver on the following:
Habulha omuthwaniri oko bino:

- ☐ Wrap the baby in warm, dry clothes, including the head and feet.
  Hira omwana omo syongimba esihuhire, esyumire, kandi neriswika omuthwe haima nebisandu.
- ☐ Put the baby in skin-to-skin contact with the mother, as shown in the picture.
  Omwana muhire oko mubiri' ko mubiri wa mama wiwe ngokokikengenie oko bisasani
- ☐ Delay the baby's first bath until after 24 hours.
  Linda eryoya olhumekeke hathali hahwa esyosaha abiri' nini [24]

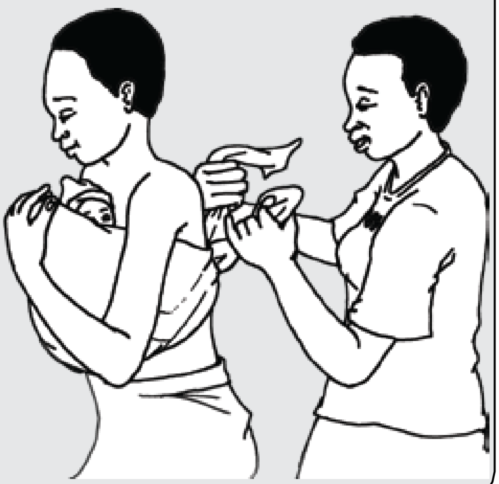

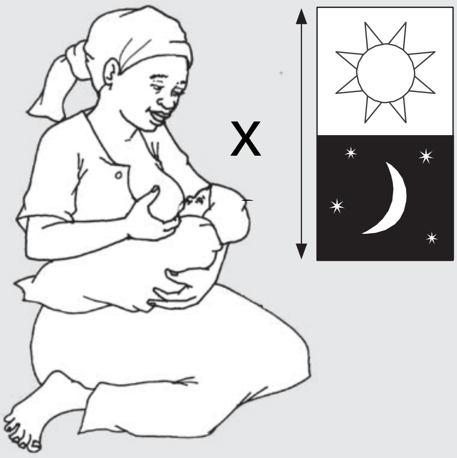

Breastfeeding exclusively.
Eryongya omwana amabere masa hathe erimuhererya ebindi bindu.

Advise the mother on the following:
Habulha mama wo mwana oko bino:

- ☐ Start breastfeeding immediately after birth.
  Tsukiraho eryongya omwana akanabuthwa.
- ☐ Feed the baby on only breast milk, on demand, at least 8 times a day.
  Ulisaye omwana amabere masa, okwisabaliwe kandi nikiniki emirundi munani [8] ekiro.
- ☐ Ensure the baby is well positioned and attached on the mother's breast.
  Langira uthi omwana anethekirwe ndeke okwibere lyamama wiwe.

Skin and cord care / Obuyojyo obwengoba haima nengundi

Advise the caregiver on the following:
Habulha omuthwaniri oko bino:

- ☐ Wash hands before handling the baby.
  Tsuka erinaba okobyalha uthali wahamba oko mwana
- ☐ Do not apply anything on the cord. Leave the cord dry and open.
  Ulikatheka akandu kosi kosi oko ngundi yo mwana. Engundi iyikalha yinum ire
  .
- ☐ Bathe the baby with clean soap and water.
  Oghoya omwana nesabuni haima namaghetse awahenirye.

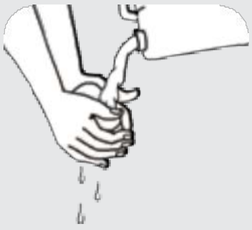

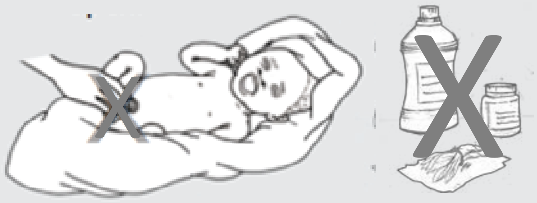

THE REPUBLIC OF UGANDA
Ministry of Health

VHT
VILLAGE HEALTH TEAM

Designed and illustrated by **Mango Tree**
www.mangotreeuganda.org

Translation and formatting by
Massachusetts General Hospital, 2012
